# Muscle Albumin as a Food

**Published:** 1901-08-31

**Authors:** 


### Hospital Clinics and Medical Progress.

### MUSCLE ALBUMIN AS A FOOD.

If, out of that complex of many influences which we include under the common phrase "the open-air treatment of consumption" we endeavour to discover wherein lies the particular factor which is the key to the success which undoubtedly attends this form of treatment in many cases, we probably shall not be far wrong in saying that it is in the improved digestive power and the resulting improvement in the "vitality" of the patient, if we may use such an expression, rather than in any specific effect of fresh air upon the disease or its bacillus. Thus the character and quantity of the food taken become matters of the greatest importance, and practically we find that at some of the best known of the sanatoria, even from the earliest stage in the treatment, every effort is made to ensure the assimilation of the largest possible amount of food.

The question was raised at Cheltenham by Dr. Forbes Ross whether the secret of success does not lie far more exclusively in this question of diet than some have imagined, and with the object of providing a food which will give the largest return for the least digestive effort, he strongly urges the use of meat albumin in the treatment of tuberculosis. Defective alimentation, he very truly says, may for all practical purposes be understood to be governed on the one hand by the digestive and assimilative powers of the sufferers, and on the other to be dependent on the degree of digestibility of the food substances supplied, this "digestibility" being calculated by comparing the amount and quality of nutriment contained in the particular food and the amount of digestive effort expended in extracting and assimilating these nutrient substances. It is the loss involved in the act of digestion that is the mischief in so many of the diets put before consumptives. Nothing, he says, can be well conceived more exhausting and distressing than a continuous effort to derive nutriment from food unsuitable in quality under circumstances of deranged function and irritative conditions of the digestive apparatus. Assimilation embraces not only the ability to take up and absorb nutriment but the power to fix it in the organism in a condition fit for use, and with this in view he regards myosin albumin or meat albumin as the most useful form of food. Comparing it with milk or casein albumin he holds that while benefits derived from the latter are rapidly dissipated under unfavourable conditions, meat albumin quickly and readily manifests its better qualities not only in the early improvement of the sufferer but in the fact that the benefit so obtained is not so easily undone.

We think there is something in all this. At the very least there is every reason to believe that, for each patient suffering from such an exhausting disease as consumption, that diet will prove the best stepping stone to improved digestive capacity which for the time being gives the most nutriment in return for the least digestive effort. Some may find this in milk, others in various meat extracts, or perhaps in some of the tasty products of the culinary art, but no one who has tried the experiment can doubt the supreme efficacy of raw meat or the freshly expressed juice of meat as a giver of strength in cases of serious disease. Of course this is no new thing. It must be thirty years ago or more that Dr. Blanc advocated the use of freshly expressed meat juice in the treatment of consumption and other exhausting diseases, and his method, which we have frequently practised, is so useful as an extemporaneous arrangement, although perhaps a little wasteful, that we may well give it here.

There must be a good bright clean fire, a gridiron, some good beef steak, a pair of sugar tongs, and a lemon squeezer. The steak is to be cut into cubes of about an inch in size; these are to be laid on the grid upon the fire and turned quickly with the sugar tongs till they are well frizzled on the outside, leaving the whole of the interior of a purple red, just turning to red. A very little experience will show when the proper point is arrived at. The pieces of meat are then taken off the grid, placed in the lemon squeezer, which should have been previously warmed, and forcibly compressed, when the juice will flow freely. It is surprising how much comes out when just the right amount of external frizzling has taken place without overdoing the interior of the bits of meat. The juice should be drank either just as it is or flavoured with pepper and salt, and it is well to give it out of a coloured glass or cup as the bright red colour of the fresh juice is repulsive to some. But to return to Dr. Ross. He says that meat albumin may be used either in a cooked state, or as a raw meat juice prepared from fresh meat and used immediately after preparation. It can be made either by mincing the meat and expressing it or by the cold extraction process with a saline solution, and this last can be used either alone as it is or it can be flavoured with port wine, spices, etc. On the other hand, meat albumin can also, he says, be utilised in a coagulated or cooked condition, and apparently with just as great benefits judging from practical results, which we venture to doubt. It seems probable that to a large extent the success of the open-air treatment of consumption hinges upon the fact that appetite being sharpened and digestion improved the patient is able to partake of those articles of food which tend to provide him with the largest amount of nourishment in return for his increasing power of dealing with them. And it is the same with the "high altitude cure" with mere "change of air," and in a less degree (owing to the lack of adequate food to meet the demand created) with sea voyages. Hence, whatever may be done in the way of providing consumptive patients with fresh air, the appetite so created and the increased digestive capacity so caused must be met by a largely increased diet of the right kind. No matter what method of treatment be adopted the matter of diet will ever remain paramount, and in such a diet muscle albumin must hold a preeminent position, seeing that, as Dr. Ross says, "it returns a maximum of nutrient for a minimum of effort." Which being interpreted means that in the diet of such cases as can take it a considerable proportion of somewhat underdone meat will be useful, while in other cases the muscle albumin must be extracted in one form or another and given as a dose.

